# Awareness, Perception, and Practice Regarding Needle-Stick Injury and Its Prevention Among Healthcare Workers in a Tertiary Care Hospital in Southern India

**DOI:** 10.7759/cureus.55820

**Published:** 2024-03-08

**Authors:** D. Anandadurai, R. Praisie, Sriandaal Venkateshvaran, Sudhir B Nelson, Manoje Thulasiram

**Affiliations:** 1 General Medicine, Velammal Medical College Hospital & Research Institute, Madurai, IND; 2 Community Medicine, Velammal Medical College Hospital & Research Institute, Madurai, IND

**Keywords:** recapping of needles, prevention, hiv, post exposure prophylaxis, hepatitis c, hepatitis b, healthcare workers, blood borne diseases, needle stick injury

## Abstract

Background

Needle stick injuries caused by various sharp and other items like hypodermic needles and intravenous cannulas are important occupational hazards for healthcare workers (HCW). Preventing injuries is the most effective way to protect workers and requires good awareness and perceptions associated with practice on a daily basis. Therefore, we did a descriptive cross-sectional study involving healthcare workers in a tertiary care hospital to find the level of awareness, perception, and practice associated with needle stick injury and its prevention.

Methodology

A descriptive cross-sectional study was conducted in a tertiary care hospital, in south India. 400 healthcare workers (doctors, nurses, technicians, and housekeeping staff) with more than one year of experience were randomly selected. An anonymous, self-reporting, semi-structured questionnaire was administered. Results are expressed in mean, standard deviation, frequency, and percentages.

Results

Out of 400 respondents, 89% had good awareness about proper disposal practices. However,44% of the participants had the misbelief that recapping needles was recommended to prevent needle stick injury (NSI), and 30.5% practiced it, with the doctors being the majority. The majority (79.8%) knew that HIV, Hepatitis B & C are blood-borne pathogens that HCWs are most commonly exposed to through needle-stick injury (NSI). However, only 49% knew that Hepatitis B has the highest risk of transmission following a needle prick. 75% were aware of the correct Hepatitis B vaccination doses. Most of the healthcare workers (89.5%) claimed to be aware of the procedure and guidelines to be followed after a needle stick injury and 96% felt that they would report NSI immediately. Awareness regarding Hepatitis C prevention was comparatively poor, with only 47% having knowledge regarding the non-availability of Hepatitis C Vaccination and& 46% about the non-availability of post-exposure prophylaxis for Hepatitis C. Among the healthcare workers, 61% were worried about having needle stick injuries but 56.5% felt that their own personal safety is secondary to patient care. Among the HCWs, 91.3% believed that needle stick injuries can be prevented. Most of the participants (93.5%) ensure that others around them take extra precautions while handling sharp/ needles. The majority, i.e. 88%, utilized a designated container for disposal of sharp items, while only 53% of respondents utilized a needle cutter or shredder. 85% of HCWs had attended specific training programs on the usage of safe devices/sharps in the preceding one-year period and 72.8% had completed the vaccination against Hepatitis B.

Conclusion

Awareness regarding needle stick injury and its prevention is patchy and not adequate across different sections of healthcare workers. Perceptions regarding needle stick injury and its prevention revealed an overall positive attitude. Practices related to needle stick injury and its prevention seem to be reasonably good except when related to recapping and waiting to dispose of until the completion of the session. Training sessions need to be tailored for specific participant groups and a 'one size fits all' philosophy cannot be followed.

## Introduction

Every field of work has its own set of occupational hazards. Among hazards specific to the healthcare industry, exposure to blood-borne infections is of particular concern. In particular, diseases caused by viruses like Hepatitis B(HBV), Hepatitis C(HCV), and Human Immunodeficiency Virus (HIV), are of concern as they may cause life-altering diseases with long-term impacts [1]. Various types of exposure can happen in a healthcare setting, which has the potential to transmit these diseases to healthcare workers(HCW). Needle-stick injuries form the vast majority of activity associated with such exposure. Additionally, injury due to other sharps like blades and IV cannulas can also contribute to occupational exposure to blood-borne diseases [2,3]. A report by the World Health Organisation at the start of the 21st century estimated that every year 1,000 HIV, 16,000 HCV, and 66,000 HBV infections occur among healthcare workers because of needle-stick injuries. In countries like India, every year, 1.6%, 4.1%, and 7.8% of healthcare workers (HCW) were estimated to be exposed to sharps injuries contaminated with HIV, Hepatitis C, and Hepatitis B, respectively. Consequently, the lifetime risk of getting infected is expected to be significant [4]. It is estimated that the career-time prevalence of occupational exposure among health workers to blood and other body fluids is around 56%, and healthcare workers in the Southeast Asian region were the most at risk of exposure [5].

Injuries from needles can happen while being involved in a variety of activities including removal of the cap, recapping needles, manipulation of needles, administering injections, during any procedure/surgery, insertion and removal of cannula, collision with another person or object, and also during clean-up and disposal [6]. Incidental punctures or cuts by contaminated needles can inject hazardous fluids into the body [7].

Preventing injuries is the most effective way to protect workers. This can only be brought out by the increase in awareness and a positive attitude toward injury prevention. Prevention also involves action taken after an exposure including post-exposure prophylaxis (PEP). PEP for HIV involves the use of multiple antiretroviral drugs and Hepatitis B involves the use of vaccine and immunoglobulin. In some healthcare settings, even when knowledge is good post-exposure practices are inadequate with many failing to report or take adequate appropriate action [8]. The level of awareness and practice also may vary significantly across different cadres of healthcare workers [9].

Hence, we conducted a cross-sectional study involving all cadres of healthcare workers in a tertiary care hospital. The objective of the study was to evaluate the awareness, perception, and practice associated with needle stick injury and its prevention.

## Materials and methods

Study design, setting, and study participants

A descriptive cross-sectional study was conducted in a tertiary care hospital, in south India from August 2023 to October 2023. Various categories of healthcare workers involved in patient care activity were selected by probability proportional to size sampling and included in this study.

Sample size

 According to Madhavan et al. [10], the prevalence of good knowledge regarding needle stick injury(NSI) was 61%, and this was considered the expected prevalence while calculating the sample size. The formula (Z_(α/2)_^2^(pq)/ d^2^) was used, where Z_(α/2)_ is 1.96, p = .61, q = 1-p, and d is the margin of error, taken at 5%. The minimum sample size was calculated as 365 samples. This was rounded up, and accordingly, the sample size was fixed at 400.

Sampling technique

Sampling was done by probability proportion to size technique. The entire healthcare workforce of the institute was divided into four categories: doctors, nurses, technicians, and housekeeping and other support staff. Based on the total number of workers in each of these categories at the start of the study, the sample was proportionally distributed. Therefore, the sample of 400 was distributed to 78 doctors, 223 nurses, 13 technicians, and 86 housekeeping/others. Workers within the group were selected as per the random sampling technique using a random number generator with their employer identification number as their assigned number.

Inclusion and exclusion criteria

All the healthcare workers who were working in this institution at the time of study were included. Those who were not willing to participate and those who had less than one year of experience at the time of data collection were excluded from the study.

Data collection tools and methods

A pretested, self-reporting semi-structured questionnaire was administered to the study participants (Appendices). The questionnaire was based on the one used by Alsabaani et al. [11] and was modified after pilot testing. The questionnaire has four parts, the first part contains sociodemographic details like age, sex, working nature, years of experience, etc. The second part has a total of 10 questions on awareness related to needle stick injury (NSI). The section started with questions on awareness regarding protocols for sharp waste disposal and recapping of the needle. It was followed by questions related to blood-borne pathogens and their risk of transmission and vaccination details like availability and dose. In addition, questions related to post-exposure prophylaxis in terms of availability, immediate action, initiations, etc. were asked. The next section is on perception, having seven questions focused on their attitude towards needle stick injury like reporting the injury and its preventive aspect and their extra precaution while handling sharps. The questionnaire also enquires about their attitude towards patient safety and post-exposure prophylaxis. The last section includes eight practices related to the risk of needle stick injury. The habit of recapping needles, bending needles, and disposing of needles in batches, the regular usage of a needle cutter, and waste disposal in specified containers were included. Questions related to the hepatitis B vaccine and serological status were also included. The questionnaire was shared with the participants selected into the sample in the form of a Google form through WhatsApp or email. The questionnaire was also translated into the local language (and back-translated) and then pilot-tested. For those who were not comfortable with English, a printed form of the questionnaire in the local language was issued. The information from the filled questionnaire was then translated and entered in Google form.

Statistical methods

Data was entered through Google form and exported to Microsoft Excel, and then analysis was done using PSPP version 1.6.2 software (Free Software Foundation, Boston, USA). Results are expressed in mean, standard deviation, frequency, and percentage.

Ethical consideration

Clearance from the Institutional Ethical Committee was obtained before the start of the study (IEC No: VMCIEC/002/2023). Informed written consent was obtained from the participants. The identity of participants was masked and individual data will not be published.

## Results

Four hundred healthcare workers from different cadres were included in the study. The mean age group of the participants was 31.3 ± 9.5 years and the age ranged from a minimum of 20 to a maximum of 67. The mean years of experience was 5.24 years. Most of the workers were female, i.e. 329 (82.3%) (Table 1).

**Table 1:** Characteristics of the sample population

Sl. No	Character	Sub variable	Frequency (Percentage)
1	Gender	Male	71 (82.3 %)
		Female	329 (17.8 %)
2	Profession	Doctor	78 (19.5%)
		Housekeeping	86 (21.5 %)
		Nurses	223 (55.8%)
		Technician	13 (3.3 %)
3	Year of Experience	1-5 Years	256 (64%)
		06-10 Years	109 (26.5%)
		11-15 Years	19 (4.8%)
		16-20 Years	10 (2.5%)
		Above 20 Years	9 (2.3%)
4	Age in years	20-29	227 (56.8%)
		30-39	93 (23.3%)
		40-49	57 (14.3%)
		50 and above	23 (5.8%)

Awareness about needle stick injury and its prevention

The awareness about various aspects of needle stick injury (NSI) and its prevention across various categories of healthcare workers (HCWs) was assessed using specific questions on NSI, disease spread, and prevention (Table 2). Out of 400 respondents, 356 (89%) knew that disposal in the proper container can reduce the risk of needle stick injury. Conversely, almost half, i.e. 176 (44%) of the participants thought that recapping needles is recommended to prevent needle stick injury.

**Table 2:** Percentage of correct responses to questions related to awareness about needle stick injury prevention * The correct answer expected is given within parentheses. ** Denotes the percentage within each group.

Sl. No	Questions^*^		Doctor n (%)**	Nurses n (%)**	Technicians n (%)**	Housekeeping n (%)**	Total n (%)**
1	Is disposal in a sharps container after performing procedures recommended to decrease the risk of needle stick injury? (Yes)		76 (97.4%)	204 (91.5%)	10 (76.9%)	66 (76.7%)	356 (89%)
2	Is recap of the needle after performing procedures recommended to decrease the risk of needle stick injury? (No)		35 (44.9%)	146 (65.5%)	11 (84.6%)	32 (37.2%)	224 (56%)
3	The correct Doses of vaccines required for full protection from Hepatitis B? (Three)		62 (79.5%)	163 (73.1%)	10 (76.9%)	65 (75.6%)	300 (75%)
4	Can Hepatitis C disease be prevented by vaccine? (No)		59 (75.6%)	114 (51%)	12 (92.3%)	27 (31.4%)	212 (53%)
5	Choose any three Blood-borne pathogens that hospital workers are most commonly exposed to when they experience needlestick injury. (Hep B, Hep C & HIV)		58 (74.4%)	176 (78.9%)	13 (100%)	72 (83.7%)	319 (79.8%)
6	Which disease carries the greatest risk of transmission in needle stick or sharp injury? (Hepatitis B)		38 (48.7%)	102 (45.7%)	9 (69.2%)	47 (54.7%)	196 (49%)
7	Are you aware of the procedure and guidelines to follow if you sustain a splash, sharp, or Needle stick injury in your workplace? (Yes)		72 (92.3%)	196 (87.9%)	13 (100%)	77 (89.5%)	358 (89.5%)
8	If you have a sharp/ needle stick injury your immediate action will be to wash with?	Soap & water	55 (70.5%)	67 (30%)	7 (53.8%)	26 (30.2%)	155 (38.8%)
		Only water	16 (20.5%)	152 (68.2%)	6 (46.2%)	49 (57%)	223 (55.8%)
9	There is currently no approved post-exposure prophylaxis for which disease? (Hepatitis C)		56 (71.8%)	81 (36.3%)	5 (38.5%)	42 (48.8%)	184 (46%)
10	Post-exposure Prophylaxis for HIV must be started within ___hours. (72 hours)		28 (35.9%)	83 (37.2%)	5 (38.5%)	33 (38.4%)	149 (37%)

When given a choice of eight diseases (Dengue, Hepatitis B, Malaria, Filariasis, Hepatitis C, Syphilis, HIV, and Leptospirosis), the majority, i.e. 319 (79.8%), knew that HIV, Hepatitis B (Hep B), and Hepatitis C (Hep C) are blood-borne pathogens that healthcare workers are most commonly exposed to through needle-stick injury. Apart from these, syphilis was the most commonly chosen - 52 (12.5%). However, only 196 (49%) knew that Hepatitis B has the highest risk of transmission following a needle prick. Three-fourths of the participants, i.e. 300 (75%), were aware of the correct number of doses of Hepatitis B vaccination.

Most of the healthcare workers - 358 (89.5%) - claimed to be aware of the procedure and guidelines to be followed after a needle stick injury. 223 (55.8%) claimed that the proper method of washing post-exposure was with water only and another 155 (38.8%) with soap and water. However, only 149 (37%) know the exact time before which, post-exposure prophylaxis for HIV needs to be started.

Knowledge regarding Hepatitis C prevention was comparatively poor, with only 212 (53%) having knowledge regarding the non-availability of Hepatitis C vaccination (particularly technicians and doctors) and 184 (46%) knowing that currently there is no approved post-exposure prophylaxis for HCV.

Perception towards needle stick injury and its prevention

The perception of HCWs concerning NSI was tested using seven questions (Table 3). 244 (61%) healthcare workers were worried about having needle stick injuries. 226 (56.5%) strongly insist patient care is more important than their own personal safety. 384 respondents (96%) felt that they would report all needle stick injuries immediately. Among the HCWs, 365 (91.3%) believed that needle stick injury can be prevented and 256 (64%) had a good attitude that even if the sharp waste is minimal and less pathogenic it should not be mixed with domestic waste. Poor attitude regarding mixing is particularly seen in housekeeping staff. 217 HCWs (54%) were worried about post-exposure prophylaxis because of various drugs and side effects and this worry is seen most among housekeeping staff and least among doctors. Most of the participants, i.e. 374 (93.5%), ensure that others around them take extra precautions while handling sharp/ needles.

**Table 3:** Responses to statements of perception towards Needle stick injury and its prevention * Positive or negative perception mentioned within parentheses. ** Denotes the percentage within the specific group of healthcare workers.

Sl. No	Statements on Perceptions*	Doctor n (%)**	Nurses n (%)**	Technicians n (%)**	Housekeeping n (%)**	Total n (%)**
1	I am worried about having a needle stick injury (+ve)	60 (76.9%)	112 (50.2%)	4 (30.8%)	68 ( 79.1%)	244 (61%)
2	Patient care is more important than my safety (-ve)	21 (26.9%)	132 (59.2%)	5 (38.5%)	68 (79.1%)	226 (56.5%)
3	I would report all sharp injuries at work immediately (+ve)	74 (94.9%)	216 (96.9%)	12 (92.3%)	82 (95.3%)	384 (96 %)
4	I think needle stick injury is preventable (+ve)	75 (96.2%)	197 (88.3%)	12 (92.3%)	81 (94.2%)	365 (91.3 %)
5	If the quantity of sharp waste is very small or if the potential of infection is less, it can be mixed with domestic waste (-ve)	11 (14.1%)	74 (33.2%)	4 (30.8%)	55 (64%)	144 (36 %)
6	I am worried about taking post-exposure prophylaxis as it involves multiple drugs or injections and side effects (-ve)	23 (29.5%)	123 (55.2%)	6 (46.2%)	65 (75.6%)	217 (54.2 %)
7	I ensure others around me take extra precautions while handling sharp/ needles (+ve)	76 (97.4%)	203 (91%)	12 (92.3%)	83 (96.5%)	374 (93.5 %)

Practice related to needle stick injury and its prevention

Practice related to NSI and its prevention including post-exposure prophylaxis and vaccination was assessed (Table 4). Six healthcare workers have been in a supervisory role for the past year and have not handled needles during the period. Hence they did not answer questions related to the handling of needles. Thus the total number of respondents to questions related to some practices was 394. Recapping of the needle is done by 120, i.e. 30.5%, of HCWs, with doctors being the majority. Also, among the 120 HCWs who recap, 57, i.e. 47.5%, recap with both hands. Almost one-third, i.e. 128 (32.5%), had the habit of bending the needle before disposal. The majority, i.e. 353 (88%), utilized a sharp container for disposal of sharp items, while, only half of the respondents, i.e. 203 (53%), utilized a needle cutter or shredder. Only 26 HCWs (6.5%) did not use either a sharp bin or a needle cutter in the disposal of needles. 210 workers (53%) had the habit of destroying the needles in batches at the completion of the procedure session.

**Table 4:** Practice related to needle stick injury and its prevention * Denotes the percentage within each group. # Total number of respondents for the related question.

Sl. No	Practice(N)^#^	Doctor n (%)*	Nurses n (%)*	Technicians n (%)*	Housekeeping n (%)*	Total^#^ n (%)*
1	Habit of recapping needles (N = 394)	46 (59.7%)	48 (21.9%)	1 (7.7%)	25 (29.4%)	120 (30.5%)
2	Habit of recapping needles with two hands (N = 120)	16 (34.8%)	20 (41.7%)	1 (100%)	20 (80%)	57 (47.5%)
3	Habit of bending needles before disposal (N = 394)	29 (37.7%)	74 (33.8%)	5 (38.5%)	20 (23.5%)	128 (32.5%)
4	Always use a needle cutter/burner, shredder, etc to dispose of a needle (N = 394)	45 (59.2%)	121 (57.4%)	6 (46.2%)	31 (37.4%)	203 (53%)
5	Always put sharp items into their assigned sharps container (N = 394)	72 (92.3%)	198 (88.8%)	9 (69.2%)	74 (86%)	353 (88.25%)
6	Collect all the needles during the procedure and dispose of them together at the end of the session (N = 394)	47 (61%)	115 (52.5%)	7 ( 53.8%)	41 (48.2%)	210 (53.3%)
7	Attended a training program on the safe usage of needles/ sharps in the last year (N = 400)	50 (64.1%)	209 (93.7%)	13 (100%)	66 (76.7%)	338 (84.5%)
8	Fully vaccinated against Hepatitis B (N = 400)	46 (59%)	180 (80.7%)	13 (100%)	52 (60.5%)	291 (72.8%)

Almost 85% (338) had attended specific training programs on the safe usage of needles/sharps in the preceding one year, where doctors were the group with the least attendance. 72.8% of HCWs had the vaccination against Hepatitis B, while another 30 among unvaccinated had an adequate titre of antibodies.

## Discussion

In our study, 176 (44%) healthcare workers (HCWs) thought that recapping needles is recommended to prevent needle stick injury (NSI). Similar findings were reported by Punia et al. (59%) and Muralidhar et al. (66%) among HCWs of Tertiary care centers in South India and New Delhi [12,13]. Though comparatively better, for some reason recapping needles is considered to be a preventive method despite various guidelines and training against it.

The majority of our study participants, 319 (79.8%) knew that HIV, Hepatitis B & C are blood-borne pathogens that healthcare workers are most commonly exposed to through needle-stick injury. Similar findings were reported by Pavithran et al. (88%) among dental professionals [14]. However, Sharma et al. reported that only 50% of HCWs were able to answer this question correctly [15]. Apart from these diseases, syphilis was the most commonly chosen disease, i.e. by 52 (12.5%). This might be due to the overlapping of blood-borne and sexually transmitted diseases. Only half, i.e. 196 (49%), knew that Hepatitis B has the highest risk of transmission following a needle prick. A study by Datar et al. revealed that only 32% of health students can do the same [16].

Most of the healthcare workers, i.e. 358 (89.5%), claimed to be aware of the procedure and guidelines to be followed after a needle stick injury, a finding similar to Aktar et al. (93%) done in coastal Karnataka [17]. More than half, i.e. 223 (55.8%), claimed that the proper method of washing post-exposure was with water only while another 155 (38.8%) claimed that it should be soap and water. Different guidelines advise post-exposure wash with water or soap and water, which might be the reason for two different answers [18,19]. Consequently, considering them together most of the HCWs were right.

Our finding regarding the prevalence of awareness regarding the non-availability of a Hepatitis C Vaccine (212, 53%) is better than that reported by Alsabaani et al. (43%) among healthcare workers in Abha City, Saudi Arabia [11]. Overall, the awareness related to Hepatitis C is comparatively low, one reason might be because it is often confused with Hepatitis B.

In our study, 244 (61%) healthcare workers were worried about having needle stick injuries. Kotwal and Taneja had a similar finding of 67% of HCWs in a hospital in New Delhi [9]. 226 (56.5%) insist patient care is more important than their own personal safety. A similar finding was reported by Yazid et al., with 40% claiming that patient care is of greater importance [20].

Both in our study and in that by Yazid et al. [20] more than 90% believed that needle stick injury could be prevented and more than 95% felt that they would report all needle stick injuries immediately. Most of the participants ensure that others around them take extra precautions while handling sharp/ needles. Therefore, the overall attitude of the HCWs is positive in most aspects and the relatively poor attitude towards segregation is somewhat ameliorated by the practice of segregation at source followed by 376 (94%) of the HCWs. 217 (54%) HCWs were worried about post-exposure prophylaxis due to various drugs and side effects.

Recapping of the needle is done by 120 (30.5%) HCWs. This is far better than the 51% reported by Madhavan et al. [10]. 32% of participants, i.e. 128 HCWs, in our study, had the habit of bending needles before disposal, a finding similar to that of Chatterjee et al. who reported finding 31.6% of nurses having this habit [21]. A far better result was reported by Punia et al. where only 13% had the habit [12]. The wrong practice of recapping is in line with the finding of a significant proportion claiming that the practice is recommended to prevent needle stick injury. Practice regarding disposal of needles after use was good with a majority, i.e. 374 (93.5%), following proper protocol. Studies by Punia et al. [12] and Kotwal and Taneja [9] reported a slightly lower proportion of 82% and 86% of respondents following proper disposal protocol, respectively.

Almost 85%, i.e. 338, of healthcare workers had attended specific training programs on the usage of safe devices/sharps in the preceding one year, where doctors were the group with the least attendance. Akthar et al. reported that 80% of their study participants had similar training [17]. Doctors were the least likely to attend training programs specific to needle stick injury. As it is part of their regular curriculum during undergraduation, there is a chance that better knowledge among doctors might be assumed leading to fewer programs focused on the topic or less attendance in such programs.

Around 20% of HCWs, i.e. 109, were not protected against Hepatitis B in our study, with the coverage being least among doctors. Similar comparatively lower coverage of doctors was observed by Punia et al., although only 7.4% lacked coverage [12]. Studies by Gurubacharya et al. [22], Singh et al. [23], and Makade et al. [8] reported lower proportions, i.e. 60%, 69%, and 69% of Hepatitis B vaccine coverage among HCWs of tertiary hospitals, respectively.

Overall, technicians are the group that stands out with better awareness, better perception, and better practice when compared to others. The better performance may be due to better training or due to the comparatively smaller sample which might not be representative of this specific group and which could be brought out by further study. However, even in this group, all three domains are not uniformly good.

Limitations

Since the data was collected using a self-reported questionnaire, there might be differences in the practice being reported and the activities in daily work. The study was also done in only one center and hence cannot be generalized to all healthcare workers.

## Conclusions

Awareness regarding needle stick injury and its prevention is patchy and not adequate across different sections of healthcare workers, similar to other studies done in Asia. There are still wrong perceptions related to recapping. Perception regarding needle stick injury and its prevention revealed an overall positive attitude. Practices related to needle stick injury and its prevention seem to be reasonably good except when related to recapping and waiting to dispose till the completion of the session.

Special programs focused on needle stick injury for all sections of healthcare workers need to be organized. 'Once size fits all' cannot be followed when implementing training programs. An initial brief assessment of specific knowledge, perception, and practice is essential before planning such programs. Then the training can be tailor-made to fit the audience. Specific subsections of knowledge need to be updated as it is shown that better awareness will lead to better perception, followed by better practice on the prevention of needle stick injury, and ultimately prevent the spread of blood-borne diseases.

## Appendices

QUESTIONNAIRE

1.  Age:  ___________in completed years                                   2. Gender:  Male / Female

3. Profession: Doctors / Nurses / Technician / Housekeeping staff

4. Years of experience in the current profession:

Questions of awareness

5. Disposal in a sharps container after performing procedures is recommended to decrease the risk of needle stick & Sharp injury. (Yes/ No)

6. Recap of the needle after performing nursing procedures is recommended to decrease the risk of needle stick injury. (Yes/ No)

7. Doses of vaccines required for full protection from Hepatitis B. (1/2/3/4/5)

8. Hepatitis C disease can be prevented by vaccine. (Yes/ No/ don’t know)

9. Choose any three Blood-borne pathogens that hospital workers are most commonly exposed to when they experience needle stick injury.  -

Dengue

Hepatitis B

Malaria

Filariasis

Hepatitis C

Syphilis

HIV

Leptospirosis

Don’t know

10. In needle stick injury, which disease carries the greatest risk of transmission. -

Dengue, Hepatitis B, Malaria, Filariasis, Hepatitis C, Syphilis, HIV, Leptospirosis, don’t know.

11. Are you aware of the procedure and guidelines to follow if you sustain a needle stick injury in your workplace? (Yes/No)

12. If you have a needle stick injury your immediate action will be to wash with

Water only

Soap and water

Betadine

Sterilium

Spirit

Peroxide

Others

13. There is currently no approved post-exposure prophylaxis for which of the following?

        HIV                      Hepatitis C                   Hepatitis B                   don’t know

14. PEP for HIV must be started within ______ hours after a needle stick injury

12        24         48              72               don’t know

Questions on perceptions

15. I am worried about having a needle stick injury. (Yes/No)

16. Patient care is more important than the my own safety. (Yes/No)

17. I would report all sharps injuries at work immediately. (Yes/No)

18. I think needle stick injury is preventable. (Yes/No)

19. If the quantity of sharp waste is very small or if the potential of infection is less, it can be mixed with domestic waste. (Yes/No)

20. I am not sure about taking Post-exposure prophylaxis as it involves multiple drugs or injections and side effects (Yes/ No)

21. I ensure others around me take extra precautions while handling sharp/ needles (Yes/No)

Questions on practice

22. Do you have the habit of recap needles before disposal? (YES/NO/ not applicable)

23. If yes, do you recap needles with 2 hands before disposal? (YES/NO)

24. Do you bend needles before disposal? (YES/NO)

25. Do you use a needle cutter/burner, shredder, etc. to dispose of a needle? (YES/NO/Occasionally/Not applicable)

26. Do you collect all the needles used during the procedure and dispose of them together at the end of the session? (Yes / No/ Occasionally)

27. Do you put sharp items into their assigned disposal container? (Always/NO/Occasionally)

28. Have you attended any training program on the safe usage of needles/ sharps in the last year last year? (YES/NO)

29. Have you been vaccinated against Hepatitis B within the last 5 years? (YES/NO)

29 a. If no, have you checked your Hepatitis B titre within the last 5 years? (YES/NO)

29 b. If yes, was it adequate? (YES/NO/ don’t know)

## References

[1] Centers for Disease Control and Prevention (CDC) (1988). Update: universal precautions for prevention of transmission of human immunodeficiency virus, hepatitis B virus, and other bloodborne pathogens in health-care settings. MMWR Morb Mortal Wkly Rep.

[2] Singru SA, Banerjee A (2008). Occupational exposure to blood and body fluids among health care workers in a teaching hospital in mumbai, India. Indian J Community Med.

[3] Shriyan A, Roche R, Annamma Annamma (2012). Incidence of occupational exposures in a tertiary health care center. Indian J Sex Transm Dis AIDS.

[4] Prüss-Üstün A, Rapiti E, Hutin Y (2003). Sharps Injuries: Global Burden of Disease from Sharps Injuries to Health-Care Workers. https://www.who.int/publications/i/item/9241562463.

[5] Mengistu DA, Dirirsa G, Mati E (2022). Global Occupational Exposure to Blood and Body Fluids among Healthcare Workers: Systematic Review and Meta-Analysis. Can J Infect Dis Med Microbiol.

[6] Mohanty A, Kabi A, Mohanty AP (2019). Health problems in healthcare workers: A review. J Family Med Prim Care.

[7] Elder A, Paterson C (2006). Sharps injuries in UK health care: a review of injury rates, viral transmission and potential efficacy of safety devices. Occup Med (Lond).

[8] Makade KG, Bhawnani D, Verma N, Dengani M (2017). Knowledge and response of health care workers after needle-stick injury in a tertiary care hospital setting in tribal Rajnandgaon, Chhattisgarh, India. Int J Res Med Sci.

[9] Kotwal A, Taneja D (2010). Health Care Workers and Universal Precautions: Perceptions and Determinants of Non-compliance. Indian J Community Med.

[10] Madhavan A, Asokan A, Vasudevan A, Maniyappan J, Veena K (2019). Comparison of knowledge, attitude, and practices regarding needle-stick injury among health care providers. J Family Med Prim Care.

[11] Alsabaani A, Alqahtani NS, Alqahtani SS (2022). Incidence, Knowledge, Attitude and Practice Toward Needle Stick Injury Among Health Care Workers in Abha City, Saudi Arabia. Front Public Health.

[12] Punia S, Nair S, Shetty RS (2014). Health Care Workers and Standard Precautions: Perceptions and Determinants of Compliance in the Emergency and Trauma Triage of a Tertiary Care Hospital in South India. Int Sch Res Notices.

[13] Muralidhar S, Singh PK, Jain RK, Malhotra M, Bala M (2010). Needle stick injuries among health care workers in a tertiary care hospital of India. Indian J Med Res.

[14] Pavithran VK, Murali R, Krishna M, Shamala A, Yalamalli M, Kumar AV (2015). Knowledge, attitude, and practice of needle stick and sharps injuries among dental professionals of Bangalore, India. J Int Soc Prev Community Dent.

[15] Sharma S, Gupta A, Arora A (2010). Knowledge, attitude and practices on needle-stick and sharps injuries in tertiary care cardiac hospital: a survey. Indian J Med Sci.

[16] Datar UV, Kamat M, Khairnar M, Wadgave U, Desai KM (2022). Needlestick and sharps' injury in healthcare students: Prevalence, knowledge, attitude and practice. J Family Med Prim Care.

[17] Akthar N, Rani U, Varma M, Palimar V (2020). Determinants of needlestick injury at coastal karnataka, india. Indian J Public Health Res Dev.

[18] World Health Organization (2010). Who Best Practices for Injections and Related Procedures Toolkit. https://iris.who.int/handle/10665/44298.

[19] Indian Council of Medical Research (2016). Hospital Infection Control Guidelines.

[20] Yazid J, Yaakub R, Yusof S, Wilandika A (2023). Needle-stick incidents among nurses: knowledge, attitude, and practices in the workplace. Asian J Environ-Behav Stud.

[21] Chatterjee DS, Halder B, Wadhwa DM, Patel MD (2019). A study to determine the training effectiveness of nurses about needle sticks injury by using kap analysis (with special reference to private hospital). Think India J.

[22] Gurubacharya DL, Mathura KC, Karki DB (2003). Knowledge, attitude, and practices among health care workers on needle-stick injuries. Kathmandu Univ Med J.

[23] Singh B, Paudel B, Kc S (2015). Knowledge and practice of health care workers regarding needle stick injuries in a tertiary care center of nepal. Kathmandu Univ Med J.

